# Socioeconomic disadvantage, air pollution, and metabolic health among women: analyses of UK Biobank and US data

**DOI:** 10.3389/fendo.2026.1948066

**Published:** 2026-09-30

**Authors:** Yuqi Ma, Mengyuan Xie, Mingming Liang, Qing Li

**Affiliations:** 1 Department of Infectious Disease and Liver Disease, The Second Hospital of Nanjing, Affiliated to Nanjing University of Chinese Medicine, Jiangsu, China; 2 Department of Obstetrics and Gynecology, Anqing Municipal Hospital, Anqing Medical Center of Anhui Medical University, Anqing, Anhui, China; 3 School of Health Management, Anhui Medical University, Hefei, Anhui, China; 4 Department of Obstetrics and Gynecology, Anqing Municipal Hospital, Anqing, Anhui, China

**Keywords:** air pollution, fatty liver index, metabolic health, socioeconomic disadvantage, women

## Abstract

**Background:**

Socioeconomic disadvantage and ambient air pollution are each associated with adverse liver and metabolic health. We examined their joint associations with liver-related and metabolic outcomes among middle-aged and older women.

**Methods:**

This multi-database observational study included UK Biobank women and US women aged 40 years or older. UK exposures were Townsend deprivation and a residential 2010 PM_2.5_–NO_2_ score; outcomes were calculated Fatty Liver Index (FLI), FLI-defined probable fatty liver, and MRI liver fat. The contextual UK main model jointly included air pollution, Townsend deprivation, age, and collapsed ethnicity; extended sensitivity models additionally included smoking, alcohol frequency, education, and walking pace. US exposures were state poverty and PM_2.5_, and the primary outcome was age-standardized obesity prevalence.

**Results:**

Among 231,525 UK women, mean FLI was 38.19 and 25.79% had FLI ≥60. In the contextual UK main model, Townsend deprivation was associated with higher FLI [β = 1.114, 95% confidence interval (CI) 1.069 to 1.159] and higher odds of FLI ≥60 [odds ratio (OR) 1.080, 95% CI 1.076 to 1.084]. The positive age-adjusted pollution associations were attenuated after Townsend adjustment (FLI β = −0.068, 95% CI −0.137 to 0.001; FLI ≥60 OR 0.999, 95% CI 0.993 to 1.004). Across 46 US jurisdictions, each one standard deviation higher poverty level was associated with 1.38 percentage points higher female obesity prevalence after PM_2.5_ and regional adjustment (95% CI 0.58 to 2.17).

**Conclusion:**

Socioeconomic disadvantage was consistently associated with poorer liver-related and metabolic health among middle-aged and older women. In contrast, the UK pollution–FLI association was not supported after concurrent adjustment for area deprivation. Social conditions and environmental exposures should be considered jointly when assessing metabolic health in women.

## 1 Introduction

Metabolic dysfunction becomes increasingly common during midlife and older age, when women carry a substantial burden of obesity, diabetes, cardiovascular disease, and steatotic liver disease. Liver fat accumulation is closely linked to systemic metabolic dysfunction and can progress to clinically important liver disease (1, 2). Social and environmental conditions may contribute to the unequal distribution of this burden.

Socioeconomic disadvantage is a central candidate explanation. Recent syntheses identify income, diet quality, education, food insecurity, and other social conditions as relevant to metabolic dysfunction-associated steatotic liver disease (MASLD) prevalence, severity, or outcomes (3, 4). More broadly, material resources, housing and employment conditions, psychosocial stress, and preventive care access are established dimensions of metabolic health inequality (5). Socioeconomic disadvantage should consequently be examined as an exposure in its own right.

Ambient air pollution may also contribute to metabolic dysfunction. Fine particulate matter and nitrogen dioxide have been linked to oxidative stress, systemic inflammation, insulin resistance, endothelial dysfunction, and altered lipid metabolism (6, 7). Epidemiological evidence has linked long-term pollution exposure to diabetes and fatty liver-related outcomes (8, 9). More recent cohort and meta-analytic studies report positive associations of PM_2.5_ with metabolic dysfunction-associated fatty liver disease (MAFLD) or nonalcoholic fatty liver disease (NAFLD), although estimates vary across exposure methods and adjustment sets (10, 11). A recent UK Biobank analysis similarly linked air pollution-related metabolic signatures with MASLD (12).

Socioeconomic disadvantage and air pollution frequently share a social and spatial distribution. Communities with fewer resources may also face higher pollution, poorer housing, occupational risks, limited food and exercise environments, chronic stress, and reduced access to healthcare (13). An association estimated for pollution with limited socioeconomic adjustment can therefore reflect variation shared with deprivation. Conversely, ambient pollution is only one feature of the broader conditions represented by socioeconomic indicators. Joint models can show whether socioeconomic associations persist after pollution adjustment and how the pollution estimate changes after socioeconomic conditions are included.

We examined the joint associations of socioeconomic disadvantage and ambient air pollution with liver-related and metabolic health among middle-aged and older women. The UK component combined Townsend deprivation, a residential PM_2.5_–NO_2_ score, Fatty Liver Index (FLI), FLI-defined probable fatty liver, and MRI liver fat. The independent US component linked state poverty and PM_2.5_ to age-standardized obesity and depression prevalence among women aged 40 years or older. We evaluated the stability of socioeconomic associations after pollution adjustment, the sensitivity of pollution estimates to sequential adjustment, and whether the socioeconomic pattern was also observed for imaging derived liver fat and in an independent US data source.

## 2 Methods

### 2.1 Study design and population

We conducted a multi-database observational study that combined an individual-level analysis in the United Kingdom with an independent state-level contextual analysis in the United States (**Figure 1** and **Table 1**). Women were the analytic population in both components, and the primary estimands were associations of socioeconomic disadvantage and air pollution with outcomes among women. The UK component used baseline and imaging data from UK Biobank. The US component used individual survey records to estimate state-level outcomes among women aged 40 years or older and linked these estimates to state poverty and ambient air pollution. It provided external triangulation of the socioeconomic pattern in women’s metabolic health. Direct replication of the individual-level UK liver phenotype was outside the scope of the US analysis.

**Figure 1:**
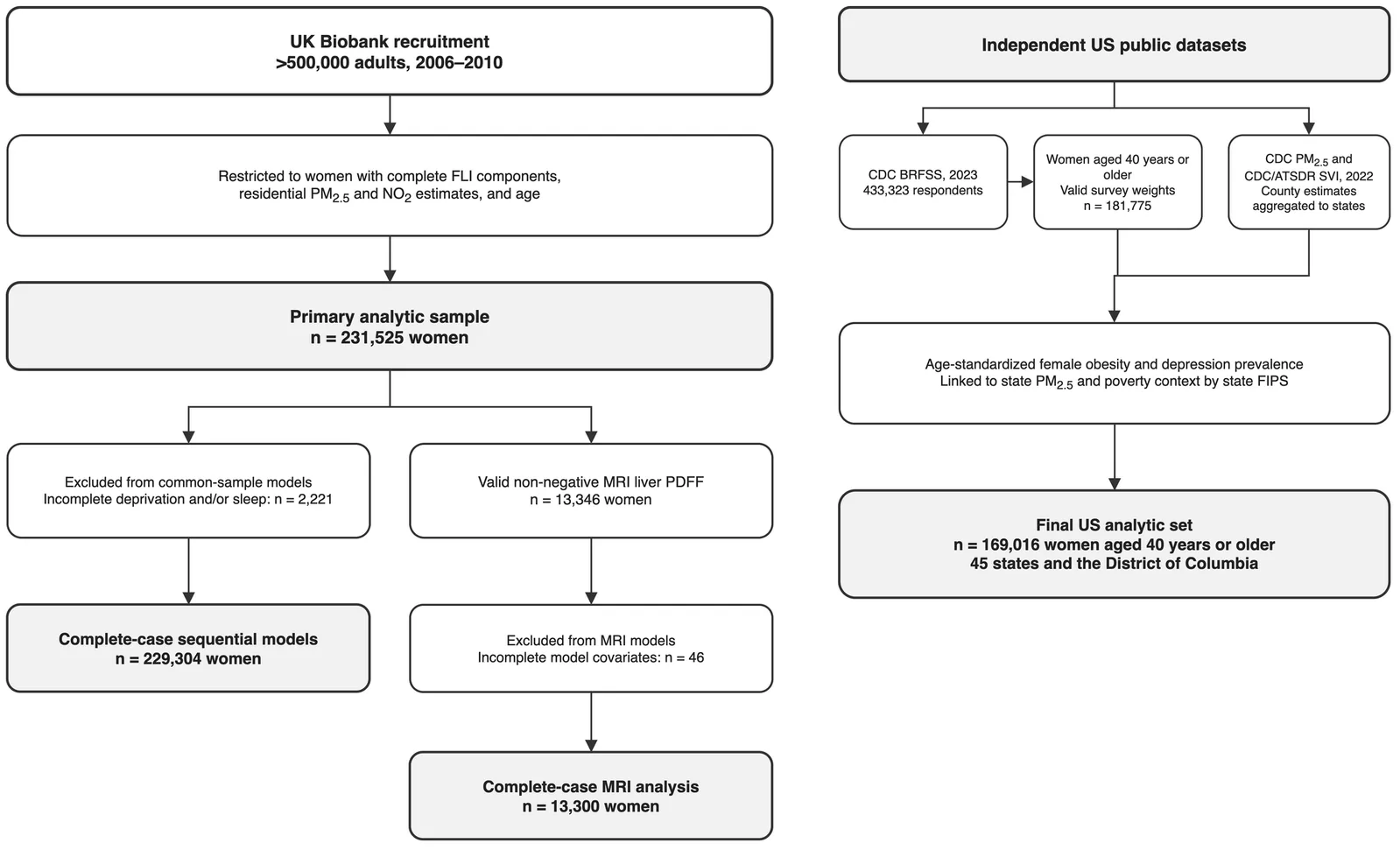
Selection of women for the UK Biobank and independent US analyses.

**Table 1:** Characteristics of the UK Biobank samples and the US female state-level analysis.

Characteristic	UK Biobank primary sample (*n* = 231,525)	UK Biobank MRI subset (*n* = 13,300)	US female state analysis (46 jurisdictions; *n* = 169,016 women)
Study population			
Data source and period	UK Biobank	UK Biobank imaging assessment	BRFSS 2023; PM_2.5_ and SVI 2022
Age eligibility	40–69 years at recruitment	40–69 years at recruitment	At least 40 years at survey
Geographic coverage	United Kingdom	United Kingdom	45 states and the District of Columbia
Survey weighted female population, *n*	—	—	82,227,168
State contextual population, *n*	—	—	307,195,459
Demographic characteristics			
Age, years	56.39 (7.99)	54.20 (7.29)	60.39 (12.31)
White background, *n* (%)	217,800 (94.07)	12,903 (97.02)	—
College or university degree, *n* (%)	70,547 (30.47)	5,897 (44.34)	—
Socioeconomic characteristics			
Townsend deprivation index	−1.39 (2.98)	−1.87 (2.70)	—
Population below 150% of the federal poverty threshold, %	—	—	20.35 (4.03)
Environmental exposures			
PM_2.5_, μg/m³	9.98 (1.05)	9.95 (1.06)	7.35 (1.26)
NO_2_, μg/m³	26.53 (7.57)	25.81 (7.31)	—
Composite air pollution score	−0.03 (1.94)	−0.15 (1.93)	—
Anthropometric and metabolic characteristics			
Body mass index, kg/m²	27.08 (5.17)	26.06 (4.55)	—
Obesity, *n* (%) or age-standardized prevalence, %	54,557 (23.56)	2,248 (16.90)	36.07 (4.36)
Waist circumference, cm	84.79 (12.52)	81.84 (11.20)	—
Triglycerides, mmol/L	1.55 (0.86)	1.41 (0.76)	—
Gamma-glutamyl transferase, U/L	30.19 (33.43)	26.65 (26.73)	—
Fatty Liver Index	38.19 (29.21); 29.63 [12.53–61.20]	30.79 (26.41); 20.90 [9.53–46.44]	—
FLI-defined probable fatty liver, *n* (%)	59,709 (25.79)	2,306 (17.34)	—
MRI liver PDFF, %	—	4.21 (4.55); 2.52 [1.91–4.25]	—
MRI liver PDFF at least 5%, *n* (%)	—	2,694 (20.26)	—
Behavioral and psychosocial characteristics			
Sleep duration, h/day	7.17 (1.11)	7.20 (0.98)	—
Current smoking, *n* (%)	20,154 (8.70)	680 (5.11)	—
Brisk walking pace, *n* (%)	88,843 (38.37)	6,560 (49.32)	—
Depressive symptoms, *n* (%) or age-standardized depression prevalence, %	69,925 (30.20)	3,406 (25.61)	25.11 (4.34)
Long-standing illness, *n* (%)	67,524 (29.16)	2,856 (21.47)	—
Good or excellent self-rated health, *n* (%)	176,354 (76.17)	11,412 (85.80)	—

Values are mean (SD), median [IQR], or *n* (%) unless otherwise indicated. US age is survey weighted. US poverty, pollution, obesity, and depression values are mean (SD) across 46 jurisdictions; obesity and depression prevalence estimates were directly age standardized among women aged 40 years or older. Dashes indicate that a measure was not collected or was not applicable. No *p*-values are shown because the columns represent different analytical datasets rather than comparison groups. FLI, Fatty Liver Index; PDFF, proton-density fat fraction; SVI, Social Vulnerability Index.

UK Biobank recruited adults aged 40 to 69 years from assessment centers across the United Kingdom between 2006 and 2010 (14). The primary sample included women with the measurements required to calculate the FLI, residential PM_2.5_ and NO_2_ estimates, and age. The main model used complete data for air pollution, Townsend deprivation, age, and ethnicity (*n* = 231,037). Extended sensitivity models added smoking, alcohol frequency, education, and walking pace (*n* = 230,340); the sleep sensitivity model included 228,632 women. An orthogonal outcome analysis included women with valid MRI liver proton density fat fraction (PDFF) and complete model covariates.

The independent US component used the 2023 Behavioral Risk Factor Surveillance System, a state-based survey of US adults (15). We restricted the survey data to women aged 40 years or older with valid sampling weights. Female obesity and depression prevalence was estimated within five age groups and directly standardized to the weighted age distribution of the eligible US female sample. County-level population-weighted PM_2.5_ estimates from the 2022 CDC Environmental Public Health Tracking data (16), and poverty estimates from the 2022 CDC/ATSDR Social Vulnerability Index (17) were aggregated to states using county population weights. Survey outcomes and contextual exposures were linked by state Federal Information Processing Standards codes. The linked analysis covered 45 states and the District of Columbia.

### 2.2 Air pollution exposure

For UK Biobank participants, annual mean residential PM_2.5_ (field 24006) and NO_2_ (field 24003) concentrations for 2010 were estimated at each participant’s residential address using land use regression models. To allow the two pollutants to be included in a single measure, PM_2.5_ and NO_2_ were standardized within the analytical sample using their means and standard deviations. The standardized values were then summed to create a continuous air pollution score, with higher values indicating higher combined exposure. The available analytical dataset did not include an authorized geographic cluster identifier that was consistent with the spatial scales used for both pollution exposure and Townsend deprivation. Therefore, we could not account for geographic clustering using cluster robust standard errors or multilevel models.

For the US analysis, annual county-level population-weighted PM_2.5_ concentrations for 2022 were aggregated to the state level using county population weights. State PM_2.5_ was standardized across linked jurisdictions so that regression coefficients represented the difference in female outcome prevalence per one standard deviation higher contextual PM_2.5_. These measures capture ambient pollution at the residential or county level. Personal exposure, occupational exposure, indoor sources, time activity patterns, and residential mobility were not available.

### 2.3 Metabolic and health outcomes

The primary UK outcome was the FLI, calculated according to the following formula (17):


$$\text{FLI}=\frac{100\times exp(L)}{1+exp(L)}$$

L=0.953×ln(triglycerides)+0.139×BMI+0.718×ln(GGT)+0.053×WC−15.745

where triglycerides were expressed in mg/dL, BMI denotes body mass index in kg/m², GGT denotes gamma-glutamyl transferase in U/L, and WC denotes waist circumference in cm. Triglyceride values reported in mmol/L were converted to mg/dL by multiplying by 88.57. FLI was analyzed as a continuous outcome, and FLI ≥60 was used to define probable fatty liver. Because BMI, waist circumference, triglycerides, and GGT are components of the FLI, these variables were not included as additional covariates in models with FLI as the outcome.

MRI-derived liver PDFF was included as an additional imaging-based outcome in the UK Biobank imaging subset. PDFF was analyzed continuously after transformation as log(1 + PDFF). It was also analyzed as a binary outcome, with PDFF ≥5% used to define hepatic steatosis. The MRI analysis was used to assess whether the patterns observed with FLI were also present when liver fat was measured directly by imaging.

The US outcomes were age-standardized state prevalence estimates of obesity and self-reported clinician diagnosed depression among women aged 40 years or older. Obesity was defined from the BRFSS BMI category as BMI of at least 30 kg/m². Depression was defined by an affirmative response to having ever been told by a health professional that the respondent had a depressive disorder. Female obesity was the external metabolic health outcome; depression was retained as a secondary psychosocial outcome.

### 2.4 Socioeconomic indicators and covariates

Socioeconomic conditions in UK Biobank were measured using the Townsend deprivation index assigned to each participant’s residential area at recruitment. The index is based on area-level unemployment, non-car ownership, non-home ownership, and household overcrowding, with higher values indicating greater deprivation.

Covariates were selected *a priori* based on previous epidemiological knowledge of socioeconomic conditions, environmental exposure, and metabolic health, together with data availability and the need to avoid inappropriate adjustment for variables that may lie on the causal pathway or form part of the outcome (18, 19). The main models included age, ethnicity, Townsend deprivation, and the air pollution score. Age and ethnicity were included as demographic factors related to metabolic health and residential patterns. Townsend deprivation and air pollution were included together because socioeconomic and environmental exposures may be correlated. Smoking, alcohol use, education, and walking pace were examined in extended sensitivity models. Previous evidence indicates that health behaviors contribute to socioeconomic differences in health, while these factors may also reflect pathways through which socioeconomic disadvantage affects metabolic health (19). Sleep duration was examined separately in an additional sensitivity model because socioeconomic conditions may influence sleep, which in turn may affect metabolic health (20, 21). BMI, waist circumference, triglycerides, and gamma glutamyl transferase were not included as covariates in FLI models because they are components of the FLI calculation.

In the US analysis, socioeconomic disadvantage was represented by the percentage of residents living below 150% of the federal poverty threshold. County estimates from the 2022 Social Vulnerability Index were aggregated to states using county population weights and standardized across the linked states and the District of Columbia. Higher standardized values indicate greater state-level poverty. In the US models, Census region was included to account for broad geographic variation shared by state poverty, ambient PM_2.5_, and female health outcomes. Covariate selection was based on epidemiological considerations and was not driven by statistical significance. Smoking, walking pace, BMI categories, and depressive symptoms were obtained from baseline UK Biobank assessments and used only in descriptive or exploratory analyses.

### 2.5 UK statistical analysis

Participant characteristics were summarized for the primary sample and MRI subset. Continuous variables are reported as mean and standard deviation or median and interquartile range when appropriate, and categorical variables are reported as number and percentage. Characteristics were also summarized across quartiles of the air pollution score. Pearson correlation quantified the association between the air pollution score and Townsend deprivation.

Linear regression was used to estimate mean differences in FLI, and logistic regression was used to estimate odds ratios (ORs) for FLI ≥60. The primary contextual model included the air pollution score, Townsend deprivation, continuous age, and collapsed ethnicity categories. Extended sensitivity models additionally included smoking, alcohol frequency, harmonized education, and walking pace. Sleep duration was added only in a separate sensitivity model because it may be influenced by socioeconomic conditions.

To examine the stability of the air pollution estimate, we fitted unadjusted, age-adjusted, and jointly adjusted contextual models. We also reported missingness for each variable and compared participants included in the final analytical sample with those excluded under the complete case approach. A targeted multiple imputation sensitivity analysis was conducted for missing Townsend deprivation and sleep duration values using predictive mean matching with five imputations. Recorded nonresponse categories for categorical variables were retained as unknown rather than imputed. Secondary analyses assessed possible nonlinear associations using natural splines and examined associations across the distribution of FLI using quantile regression. We also tested interactions with age and walking pace and analyzed FLI using three categories: <30, 30 to <60, and ≥60. As a sensitivity analysis, the corresponding UK Biobank models were also fitted in the full eligible population without restriction to women. This analysis was used only to assess the robustness of the main pattern.

### 2.6 US statistical analysis

The BRFSS complex survey design was specified using the final sampling weight, stratum, and primary sampling unit. Within each jurisdiction, survey weighted obesity and depression prevalence was estimated separately for women aged 40–49, 50–59, 60–69, 70–79, and at least 80 years. Direct standardization then applied the national weighted age distribution of eligible women to the five age specific estimates. Women with missing outcome data were excluded only from the corresponding outcome estimate.

County PM_2.5_ and poverty values were aggregated to states using county population weights and linked to the standardized female outcomes. Separate state-level linear regression models were fitted for obesity and depression prevalence. The main model included standardized PM_2.5_, standardized poverty, and Census region; the coefficients represented percentage point differences in age-standardized female prevalence per one standard deviation higher contextual exposure. A model containing PM_2.5_ and poverty without region adjustment showed the effect of adding broad regional structure. HC1 heteroscedasticity consistent confidence intervals (CIs) were used, and each jurisdiction received equal weight in the primary analysis. Population-weighted models, and models containing a PM_2.5_ by poverty product term, were treated as sensitivity or exploratory analyses. These state-level models estimated ecological contextual associations and were not interpreted as individual-level exposure effects.

All analyses were performed using R version 4.5.1 (R Foundation for Statistical Computing, Vienna, Austria). Tests were two sided, and estimates are reported with 95% CIs. A *p*-value below 0.05 was used as a descriptive threshold for statistical evidence.

## 3 Results

### 3.1 Study populations

The primary UK Biobank analysis included 231,525 women with a mean age of 56.4 years. The mean FLI was 38.19 (SD 29.21), and the median was 29.63 (IQR 12.53–61.20). Overall, 59,709 women (25.79%) had FLI ≥60, indicating probable fatty liver (**Table 1**). The primary contextual model included 231,037 women with complete data for the variables included in that model. Missing data were limited, with 0.11% of participants missing Townsend deprivation values and 0.85% missing sleep duration data (**Figure 1**).

The MRI subset included 13,300 women and was younger, less deprived, and metabolically healthier than the primary sample. Mean age was 54.2 years, mean Townsend deprivation index was −1.87, and 2,694 women, or 20.3%, had liver PDFF of at least 5%. The BRFSS restriction identified 181,775 women aged 40 years or older; 169,016 were represented in the 45 states and District of Columbia with complete contextual linkage (**Figure 1**). Their survey weighted mean age was 60.4 years. Across the 46 linked jurisdictions, mean PM_2.5_ was 7.35 micrograms per cubic meter, mean poverty prevalence was 20.35%, and mean age-standardized female obesity prevalence was 36.07% (**Table 1**).

### 3.2 Socioeconomic disadvantage

In the main model, greater Townsend deprivation was associated with poorer liver-related metabolic health after adjustment for the air pollution score, continuous age, and ethnicity. Each one unit increase in the Townsend deprivation index was associated with a 1.114 point higher FLI (95% CI, 1.069 to 1.159) and an 8.0% increase in the odds of FLI ≥60 (OR 1.080, 95% CI 1.076 to 1.084). These associations were precise and consistent across the continuous and binary FLI outcomes (**Table 2**).

**Table 2:** Associations of socioeconomic deprivation and air pollution with liver-related metabolic outcomes in 231,037 UK Biobank women.

Outcome	Model and term	Effect measure	Estimate	Conf low	Conf high	*p*-value
FLI	Age-adjusted air pollution score	β	0.842	0.781	0.903	<0.001
FLI	Contextual Townsend deprivation index	β	1.114	1.069	1.159	<0.001
FLI	Contextual air pollution score	β	−0.068	−0.137	0.001	0.052
FLI ≥60	Age-adjusted air pollution score	Odds ratio	1.062	1.057	1.067	<0.001
FLI ≥60	Contextual Townsend deprivation index	Odds ratio	1.080	1.076	1.084	<0.001
FLI ≥60	Contextual air pollution score	Odds ratio	0.999	0.993	1.004	0.624

The fully adjusted model includes the air pollution score, Townsend deprivation, continuous age, and collapsed ethnicity.

The imaging results showed the same socioeconomic direction. In models including the pollution score, age, and sleep duration, each one unit higher Townsend index was associated with a 0.009 higher log-transformed liver PDFF (95% CI 0.005 to 0.013) and greater odds of liver PDFF of at least 5% (OR 1.032, 95% CI 1.014 to 1.049; **Supplementary Table S5**). Socioeconomic disadvantage was therefore associated with both the composite metabolic index and directly measured liver fat.

### 3.3 Pollution estimates

The air pollution score and Townsend deprivation were moderately correlated in the UK contextual sample (Pearson *r* = 0.477), indicating that women with higher modeled residential pollution exposure also tended to live in more deprived areas (**Figure 2**).

**Figure 2:**
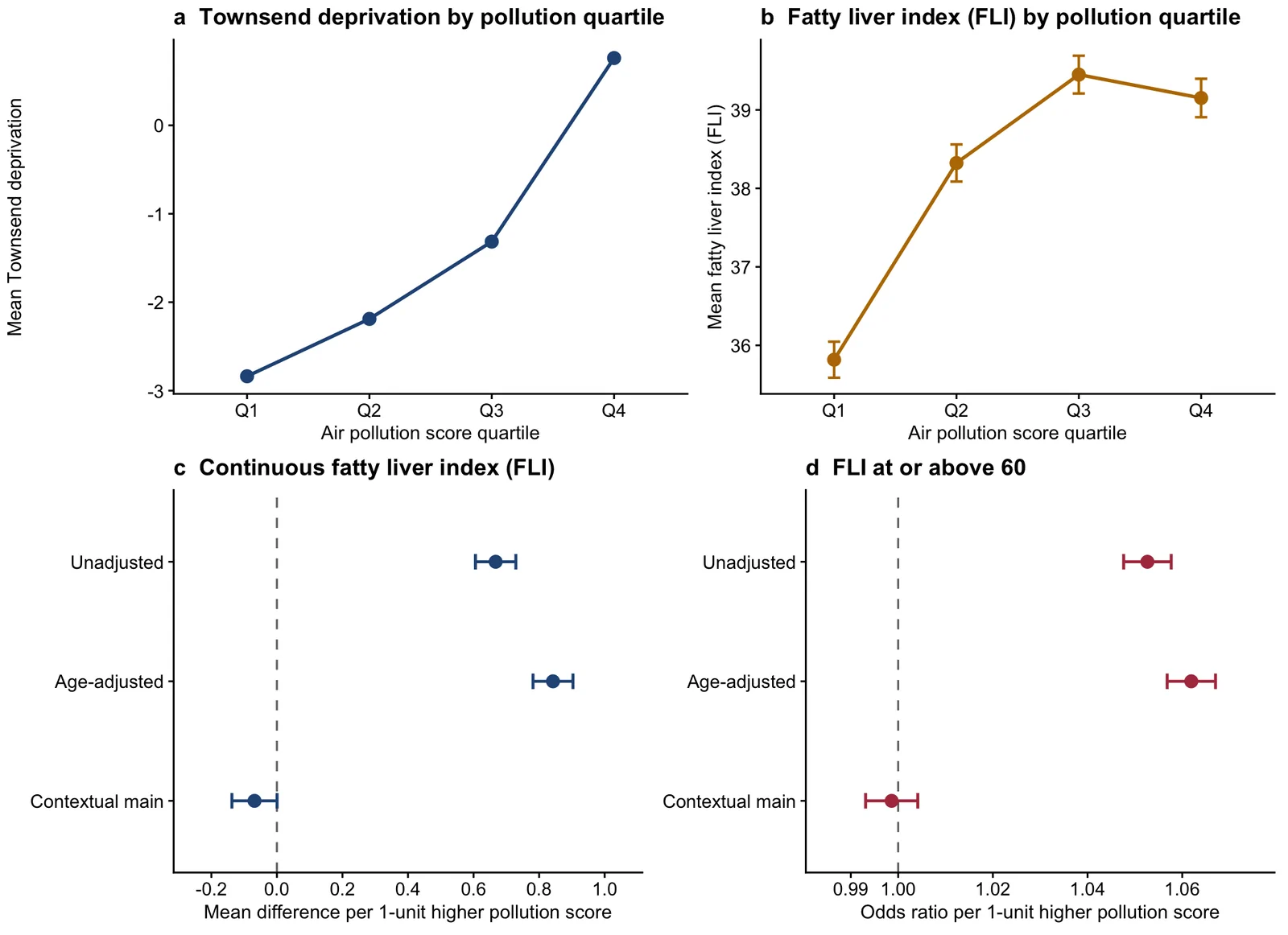
Air pollution score, Townsend deprivation, and FLI. **(A, B)** Means by pollution-score quartile; error bars show 95% confidence intervals. **(C, D)** Per-unit pollution-score estimates across models.

In age-adjusted models, each one unit increase in the air pollution score was associated with a 0.842 point higher FLI (95% CI, 0.781 to 0.903) and higher odds of FLI ≥60 (OR 1.062, 95% CI 1.057 to 1.067). After additional adjustment for Townsend deprivation and ethnicity, these associations were substantially attenuated. The coefficient for continuous FLI was −0.068 (95% CI −0.137 to 0.001; *p* = 0.052), and the OR for FLI ≥60 was 0.999 (95% CI 0.993 to 1.004; *p* = 0.624) (**Table 2**).

Targeted multiple imputation sensitivity analyses produced nearly identical estimates for both outcomes (continuous FLI: β = −0.067; 95% CI −0.136 to 0.002; FLI ≥60: OR 0.999; 95% CI 0.993 to 1.004). These results indicate that the findings from the main contextual model were robust to the small amount of missing information on Townsend deprivation and sleep duration.

### 3.4 US female outcomes

The independent US analysis showed a similar adverse socioeconomic pattern for the metabolic outcome (**Table 3**). In the model containing state poverty and PM_2.5_, each one standard deviation higher poverty level was associated with 1.837 percentage points higher age-standardized female obesity prevalence (95% CI 0.534 to 3.139). After Census region adjustment, the poverty estimate was 1.379 percentage points (95% CI 0.585 to 2.174). The PM_2.5_ estimate was −0.020 percentage points (95% CI −1.001 to 0.960).

**Table 3:** Associations of state poverty and PM_2.5_ with age-standardized obesity and depression prevalence among US women aged 40 years or older.

Outcome	Model	PM_2.5_-adjusted mean difference 95ci	Poverty-adjusted mean difference 95 CI
Female obesity prevalence	PM_2.5_ and poverty	1.091 (−0.327 to 2.509)	1.837 (0.534 to 3.139)
Female obesity prevalence	PM_2.5_, poverty, and Census region	−0.020 (−1.001 to 0.960)	1.379 (0.585 to 2.174)
Female obesity prevalence	PM_2.5_, poverty, and Census region; population weighted	−0.151 (−1.560 to 1.258)	1.578 (0.700 to 2.456)
Female depression prevalence	PM_2.5_ and poverty	−0.374 (−1.678 to 0.930)	1.402 (−0.255 to 3.059)
Female depression prevalence	PM_2.5_, poverty, and Census region	−0.453 (−2.052 to 1.146)	1.533 (−0.232 to 3.298)
Female depression prevalence	PM_2.5_, poverty, and Census region; population weighted	−1.503 (−3.115 to 0.110)	1.823 (0.069 to 3.577)

The population-weighted region-adjusted poverty estimate was 1.578 percentage points (95% CI 0.700 to 2.456). The corresponding PM_2.5_ interval included the null. Poverty estimates for female depression were positive and less precise: 1.533 percentage points in the equal jurisdiction model (95% CI −0.232 to 3.298) and 1.823 percentage points in the population-weighted model (95% CI 0.069 to 3.577).

### 3.5 Imaging and sensitivity analyses

In the MRI analysis, Townsend deprivation was associated with higher continuous liver PDFF and greater odds of liver PDFF of at least 5% (**Figure 3**; **Supplementary Table S5**). The pollution score coefficient for log-transformed liver PDFF was −0.0052 (95% CI −0.0107 to 0.0002), and the OR for liver PDFF of at least 5% was 0.986 (95% CI 0.962 to 1.011).

**Figure 3:**
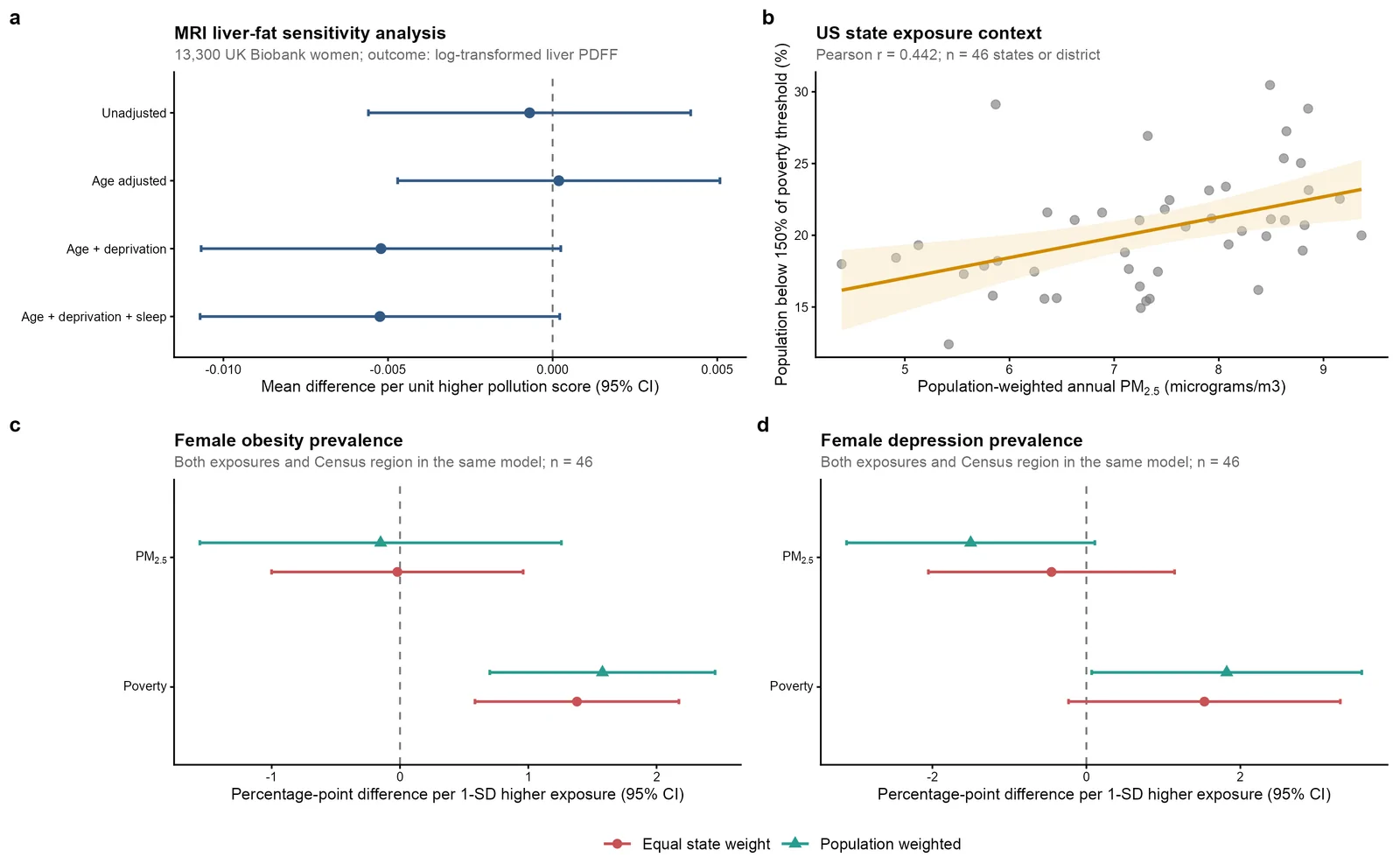
MRI outcome analysis and independent US female contextual triangulation. **(a)** MRI liver-fat sensitivity analysis in 13,300 UK Biobank women, with log-transformed liver proton density fat fraction (PDFF) as the outcome. **(b)** Association between population-weighted annual PM2.5 concentrations and the proportion of the population living below 150% of the poverty threshold across 46 US states or districts. **(c)** Associations of PM2.5 and poverty with female obesity prevalence, using equal-state and population-weighted estimates. **(d)** Associations of PM2.5 and poverty with female depression prevalence, using equal-state and population-weighted estimates.

Exploratory UK models suggested variation in the pollution slope across Townsend levels. In the US analysis, poverty by PM_2.5_ interaction intervals included the null for both female obesity and depression in equal jurisdiction and population-weighted models. The interaction results did not show a stable direction across outcomes or analytical scales.

The full population UK sensitivity analysis did not materially change the direction or interpretation of the main findings. Complete sequential, interaction, nonlinear, quantile, subgroup, multinomial, imaging, and population-weighted results are reported in the **Supplementary Material**.

## 4 Discussion

Across two analytical settings, socioeconomic disadvantage remained associated with poorer liver-related and metabolic health among women after ambient air pollution was included in the models. In 231,037 UK Biobank women, higher Townsend deprivation was associated with higher FLI and greater odds of FLI ≥60 after adjustment for the combined residential pollution score, continuous age, and ethnicity. In independent US data, higher state poverty was associated with higher age-standardized obesity prevalence among women aged 40 years or older after adjustment for PM_2.5_ and Census region. The depression estimate was directionally similar but less precise.

The pollution estimates were less stable. In the UK analysis, the positive age-adjusted association of the air pollution score with FLI and probable fatty liver changed direction after Townsend deprivation was included. In the US analysis, the region-adjusted PM_2.5_ coefficient for female obesity approached the null after poverty was included. The MRI analysis did not reproduce the primary FLI pollution association, and interaction estimates did not show a consistent direction across the UK and US analyses. The combined evidence therefore showed a clearer socioeconomic pattern in women’s health than a single, transportable pollution association.

Socioeconomic disadvantage can influence metabolic health through pathways that extend beyond ambient pollution. Evidence on social determinants and MASLD identifies diet quality, income, food insecurity, education, and access to care as relevant dimensions (3). Social class is also associated with diet quality (22), and social conditions influence diabetes prevention and management (23). These observations provide plausible context for the socioeconomic associations that remained after measured pollution was included.

The regression coefficients cannot identify which pathway is responsible. Townsend deprivation is an area-level indicator, and state poverty is a broader contextual measure. Both summarize correlated conditions and omit important individual dimensions of socioeconomic position. The consistent direction across FLI, MRI liver fat, and US female obesity therefore supports a persistent socioeconomic pattern, not a single causal mechanism.

The positive UK pollution association in the age-adjusted model was observed where pollution and deprivation were moderately correlated. Pollution exposure is frequently patterned by socioeconomic disadvantage (13, 24), and residential segregation can also structure environmental inequity (25). A minimally adjusted pollution coefficient can therefore combine environmental and social contrasts. Once Townsend deprivation and ethnicity were included, the coefficient compared women with similar measured deprivation, age, and ethnicity.

The inverse conditional coefficient is not evidence that air pollution improves metabolic health. Interpretation of coefficients for correlated predictors requires care (26). Air pollution epidemiology is also vulnerable to residual confounding and exposure measurement error (27). Proxy exposure measures and spatial scale can further alter precision and bias (26, 28). These results indicate substantial model sensitivity, and the positive age-adjusted association was not robust to socioeconomic adjustment.

Positive associations between long-term pollution exposure and fatty liver or related metabolic outcomes have been reported in a Chinese MAFLD cohort (9) and another longitudinal MAFLD study (11). A UK Biobank metabolomic analysis also reported pollution-related signatures associated with MASLD (12), and a recent meta-analysis synthesized positive pollution–NAFLD associations (10). Our age-adjusted findings were directionally compatible with that literature, whereas the socioeconomic sensitivity and lack of MRI confirmation limit direct comparison.

The US analysis provided complementary evidence from a different population and data system. State poverty was associated with higher age-standardized obesity prevalence among women aged 40 years or older after adjustment for PM_2.5_ and Census region, and the association was retained when jurisdictions were weighted by population. The depression estimate was positive but less precise. This comparison uses triangulation across data structures, not numerical replication of the UK coefficients (29).

The US outcomes were calculated from individual BRFSS records, but the exposures and final unit of analysis were states. Ecological associations can differ from individual-level associations because the exposure contrast and the meaning of the outcome vary with the unit of analysis (30). The US results should therefore be described as contextual triangulation, not external validation of an individual-level liver outcome. The two analyses are complementary rather than replicative. The UK Biobank analysis provides the primary individual-level evidence on socioeconomic disadvantage, ambient air pollution, and liver-related outcomes, whereas the US analysis examines whether a broader socioeconomic pattern is also observable for metabolic health in a different population and data structure. Because the socioeconomic indicators, pollution measures, spatial scales, and outcomes are not directly equivalent, consistency between the two settings should be interpreted only at the level of the broader association between socioeconomic disadvantage and adverse metabolic health in women. The US obesity findings do not provide independent confirmation of the liver-specific associations observed in the UK analysis.

The lack of association with MRI liver PDFF is an important qualification. FLI combines BMI, waist circumference, triglycerides, and gamma glutamyl transferase and therefore reflects a broader adiposity-related metabolic phenotype. MRI PDFF measures liver fat more directly, although the imaging subset was smaller, healthier, less deprived, and assessed at a later time. Failure to reproduce the pollution association with MRI suggests that the primary FLI pattern should not be interpreted as direct evidence of an association with liver fat itself. The difference between FLI and MRI results may reflect outcome composition, selection into imaging, exposure timing, and limited statistical precision. Several mechanisms may contribute. The findings support clear distinction between screening indices and imaging confirmed steatosis. Future studies should combine repeated pollution assessment, detailed socioeconomic measures, and imaging-derived liver outcomes in independent individual-level cohorts.

The results support joint consideration of environmental and socioeconomic conditions. In these analyses, the socioeconomic association remained after measured pollution was included, indicating that the observed socioeconomic pattern was not explained by the pollution measures available in this study. Environmental epidemiology may benefit from reporting correlations between pollution and socioeconomic indicators, comparing sequential models in common analytical samples, and clarifying how covariate adjustment changes the interpretation of exposure coefficients (31). These findings also support considering socioeconomic context alongside environmental exposures when examining metabolic health inequalities, while the observational design does not permit conclusions about the effects of specific policy interventions.

Strengths include the consistent focus on women across both data sources, the large individual-level UK sample, use of the same participants across sequential models, estimation of the deprivation coefficient after pollution adjustment, continuous and binary metabolic outcomes, and an orthogonal MRI analysis. The independent US component used BRFSS individual records and its complex survey design to estimate age-standardized outcomes among women, with regional adjustment, population weighting, and interaction analyses. It used no UK Biobank records and showed that the socioeconomic pattern for female obesity was observable in a different population and data system.

Several limitations should be considered. First, the UK analysis was observational and predominantly cross-sectional; it cannot establish causal effects. Second, FLI is a surrogate rather than imaging or histological confirmation. Third, residential pollution estimates do not capture indoor exposure, occupational exposure, daily mobility, or exposure changes over time. Fourth, Townsend is area based and cannot represent all dimensions of individual socioeconomic position. No geographic identifier was available at a spatial scale compatible with both exposure and deprivation assignment. Complete case analyses may introduce selection bias, although targeted multiple imputation results were similar. The UK and US analyses also differed in their socioeconomic indicators, pollution metrics, spatial resolution, analytical units, and outcome definitions. In particular, the UK analysis used the Townsend deprivation index and a residential PM_2.5_–NO_2_ score with liver-related individual outcomes, whereas the US analysis used state-level poverty and PM_2.5_ with age-standardized obesity prevalence. These differences preclude direct numerical replication or external validation across the two datasets. Accordingly, the US analysis should be interpreted as complementary contextual triangulation of a broader socioeconomic–metabolic health pattern rather than as confirmation of the UK liver-specific findings.

## 5 Conclusion

Among middle-aged and older women, greater socioeconomic disadvantage was consistently associated with less favorable liver-related and metabolic health across the UK and US analyses. Air pollution showed a positive association with FLI in the initial UK models. This association was substantially attenuated after accounting for deprivation. The findings highlight the importance of socioeconomic context in studies of environmental and metabolic health. They represent observational associations and should not be interpreted as evidence of causal effects.

## Data Availability

The data analyzed in this study is subject to the following licenses/restrictions: Individual-level UK Biobank data are available to eligible researchers through the UK Biobank access process under its standard data-use conditions. This study used UK Biobank Application Number 151711. The external validation used publicly available CDC Environmental Public Health Tracking, CDC PLACES, and CDC/ATSDR Social Vulnerability Index data. The processed county-level analysis data and model outputs used for the figures are included in the accompanying analysis package. Requests to access these datasets should be directed to Mingming Liang: liamg9036@ahmu.edu.cn.
