# Supplementary material for: Socioeconomic disadvantage, air pollution, and metabolic health among women: analyses of UK Biobank and US data
Source: Front Endocrinol (Lausanne). 2026 Sep 30;17:1948066. doi: 10.3389/fendo.2026.1948066 (PMC13661437; doi:10.3389/fendo.2026.1948066)
Supplement: Supplementary file 1 [file SupplementaryFile1.docx]

**Supplementary Material:**

Manuscript: Socioeconomic disadvantage, air pollution, and metabolic health among women: analyses of UK Biobank and US data

- Supplementary Table S1. FLI sequential associations of APS and Townsend deprivation in the contextual UK sample.
- Supplementary Table S2. Contextual-model APS associations across FLI quantiles.
- Supplementary Table S3. Formal FLI interaction estimates for age and walking pace.
- Supplementary Table S4. Contextual multinomial APS associations with FLI categories.
- Supplementary Table S5. Contextual MRI liver-PDFF and targeted multiple-imputation sensitivity results.
- Supplementary Table S6. Full independent US state models for age-standardized obesity and depression prevalence among women aged 40 years or older.
- Supplementary Table S7. FLI APS slopes across Townsend deprivation levels.
- Supplementary Figure S1. Contextual-model association of APS with FLI; shading shows 95% CI.
- Supplementary Figure S2. Contextual multinomial APS associations with FLI categories (reference: FLI <30).
- Supplementary Figure S3. FLI APS slopes across Townsend deprivation levels.
- Supplementary Figure S4. FLI secondary analyses.

**Supplementary Table S1. FLI sequential associations of APS and Townsend deprivation in the contextual UK sample.**

| **Outcome** | **Model** | **Exposure** | **Effect** | **Estimate** | **95% CI** | **P value** | **Covariates** |
| --- | --- | --- | --- | --- | --- | --- | --- |
| FLI | Unadjusted | APS | β | 0.667 | 0.606 to 0.729 | <0.001 | None |
| FLI | Age adjusted | APS | β | 0.842 | 0.781 to 0.903 | <0.001 | Age |
| FLI | Contextual main | APS | β | -0.068 | -0.137 to 0.001 | 0.052 | Townsend, age, ethnicity |
| FLI | Contextual main | TOWNSEND | β | 1.114 | 1.069 to 1.159 | <0.001 | Townsend, age, ethnicity |
| FLI ≥60 | Unadjusted | APS | OR | 1.053 | 1.048 to 1.058 | <0.001 | None |
| FLI ≥60 | Age adjusted | APS | OR | 1.062 | 1.057 to 1.067 | <0.001 | Age |
| FLI ≥60 | Contextual main | APS | OR | 0.999 | 0.993 to 1.004 | 0.624 | Townsend, age, ethnicity |
| FLI ≥60 | Contextual main | TOWNSEND | OR | 1.080 | 1.076 to 1.084 | <0.001 | Townsend, age, ethnicity |

**Supplementary Table S2. Contextual-model APS associations across FLI quantiles.**

| **FLI quantile** | **APS β** | **95% CI** | **P value** | **n** | **Covariates** |
| --- | --- | --- | --- | --- | --- |
| 10th percentile | -0.055 | -0.081 to -0.029 | <0.001 | 231037 | Townsend, age, ethnicity |
| 25th percentile | -0.051 | -0.097 to -0.005 | 0.030 | 231037 | Townsend, age, ethnicity |
| 50th percentile | -0.050 | -0.152 to 0.052 | 0.337 | 231037 | Townsend, age, ethnicity |
| 75th percentile | 0.076 | -0.076 to 0.229 | 0.326 | 231037 | Townsend, age, ethnicity |
| 90th percentile | 0.067 | -0.031 to 0.165 | 0.182 | 231037 | Townsend, age, ethnicity |

**Supplementary Table S3. Formal FLI interaction estimates for age and walking pace.**

| **Analysis** | **Interaction term** | **Effect** | **Estimate** | **SE** | **95% CI** | **P value** |
| --- | --- | --- | --- | --- | --- | --- |
| Age interaction | APS × age (≥60 vs <60) | β | -0.034 | 0.059 | -0.150 to 0.081 | 0.557 |
| Walking-pace interaction | APS × walking pace (linear) | β | -0.418 | 0.352 | -1.107 to 0.271 | 0.235 |
| Walking-pace interaction | APS × walking pace (quadratic) | β | -0.558 | 0.264 | -1.075 to -0.041 | 0.034 |
| Walking-pace interaction | APS × walking pace (cubic) | β | 0.028 | 0.124 | -0.215 to 0.271 | 0.823 |

**Supplementary Table S4. Contextual multinomial APS associations with FLI categories.**

| **FLI category** | **Odds ratio** | **95% CI** | **P value** | **n** | **Reference** |
| --- | --- | --- | --- | --- | --- |
| FLI 30 to <60 | 0.995 | 0.989 to 1.001 | 0.131 | 231037 | FLI <30 |
| FLI ≥60 | 0.997 | 0.991 to 1.003 | 0.345 | 231037 | FLI <30 |

**Supplementary Table S5. Contextual MRI liver-PDFF and targeted multiple-imputation sensitivity results.**

| **Outcome** | **Analysis/model** | **Exposure** | **Effect** | **Estimate** | **95% CI** | **P value** |
| --- | --- | --- | --- | --- | --- | --- |
| log1p liver PDFF | Contextual MRI model | APS | β | -0.005 | -0.011 to 0.000 | 0.060 |
| log1p liver PDFF | Contextual MRI model | Townsend | β | 0.009 | 0.005 to 0.013 | <0.001 |
| PDFF ≥5% | Contextual MRI model | APS | OR | 0.986 | 0.962 to 1.011 | 0.266 |
| PDFF ≥5% | Contextual MRI model | Townsend | OR | 1.032 | 1.014 to 1.049 | 0.001 |
| FLI | Targeted MI | APS | β | -0.067 | -0.136 to 0.002 | 0.056 |
| FLI ≥60 | Targeted MI | APS | OR | 0.999 | 0.993 to 1.004 | 0.656 |

**Supplementary Table S6. Full independent US state models for age-standardized obesity and depression prevalence among women aged 40 years or older.**

| **outcome** | **model** | **weighting** | **pm25 mean difference 95ci p** | **poverty mean difference 95ci p** | **interaction mean difference 95ci p** | **n jurisdictions** | **unweighted women aged 40plus** |
| --- | --- | --- | --- | --- | --- | --- | --- |
| Female obesity prevalence | PM_2.5_ only | Equal state weight | 1.902 (0.675 to 3.130); P=0.002 | Not estimated | Not estimated | 46 | 169016 |
| Female obesity prevalence | PM_2.5_ and poverty | Equal state weight | 1.091 (-0.327 to 2.509); P=0.132 | 1.837 (0.534 to 3.139); P=0.006 | Not estimated | 46 | 169016 |
| Female obesity prevalence | PM_2.5_, poverty, and Census region | Equal state weight | -0.020 (-1.001 to 0.960); P=0.968 | 1.379 (0.585 to 2.174); P<0.001 | Not estimated | 46 | 169016 |
| Female obesity prevalence | PM_2.5_ x poverty and Census region | Equal state weight | 0.060 (-0.846 to 0.965); P=0.897 | 1.390 (0.733 to 2.046); P<0.001 | 0.758 (-0.029 to 1.545); P=0.059 | 46 | 169016 |
| Female obesity prevalence | PM_2.5_ only | Population weighted | 0.883 (-2.632 to 4.399); P=0.622 | Not estimated | Not estimated | 46 | 169016 |
| Female obesity prevalence | PM_2.5_ and poverty | Population weighted | -0.101 (-3.733 to 3.532); P=0.957 | 2.723 (0.493 to 4.953); P=0.017 | Not estimated | 46 | 169016 |
| Female obesity prevalence | PM_2.5_, poverty, and Census region | Population weighted | -0.151 (-1.560 to 1.258); P=0.834 | 1.578 (0.700 to 2.456); P<0.001 | Not estimated | 46 | 169016 |
| Female obesity prevalence | PM_2.5_ x poverty and Census region | Population weighted | -0.109 (-1.585 to 1.368); P=0.885 | 1.470 (0.572 to 2.369); P=0.001 | 0.821 (-0.880 to 2.522); P=0.344 | 46 | 169016 |
| Female depression prevalence | PM_2.5_ only | Equal state weight | 0.246 (-0.809 to 1.301); P=0.648 | Not estimated | Not estimated | 46 | 169016 |
| Female depression prevalence | PM_2.5_ and poverty | Equal state weight | -0.374 (-1.678 to 0.930); P=0.574 | 1.402 (-0.255 to 3.059); P=0.097 | Not estimated | 46 | 169016 |
| Female depression prevalence | PM_2.5_, poverty, and Census region | Equal state weight | -0.453 (-2.052 to 1.146); P=0.579 | 1.533 (-0.232 to 3.298); P=0.089 | Not estimated | 46 | 169016 |
| Female depression prevalence | PM_2.5_ x poverty and Census region | Equal state weight | -0.348 (-1.922 to 1.226); P=0.665 | 1.546 (-0.131 to 3.224); P=0.071 | 0.994 (-0.510 to 2.499); P=0.195 | 46 | 169016 |
| Female depression prevalence | PM_2.5_ only | Population weighted | -0.106 (-2.102 to 1.890); P=0.917 | Not estimated | Not estimated | 46 | 169016 |
| Female depression prevalence | PM_2.5_ and poverty | Population weighted | -0.755 (-2.884 to 1.374); P=0.487 | 1.797 (-0.088 to 3.682); P=0.062 | Not estimated | 46 | 169016 |
| Female depression prevalence | PM_2.5_, poverty, and Census region | Population weighted | -1.503 (-3.115 to 0.110); P=0.068 | 1.823 (0.069 to 3.577); P=0.042 | Not estimated | 46 | 169016 |
| Female depression prevalence | PM_2.5_ x poverty and Census region | Population weighted | -1.415 (-2.985 to 0.156); P=0.077 | 1.600 (0.006 to 3.194); P=0.049 | 1.695 (-0.147 to 3.537); P=0.071 | 46 | 169016 |

**Supplementary Table S7. FLI APS slopes across Townsend deprivation levels.**

| **Townsend level** | **Townsend value** | **APS β** | **95% CI** | **P value** |
| --- | --- | --- | --- | --- |
| 10th percentile | -4.560 | 0.677 | 0.578 to 0.777 | <0.001 |
| Median | -2.190 | 0.220 | 0.146 to 0.295 | <0.001 |
| 90th percentile | 3.140 | -0.807 | -0.906 to -0.708 | <0.001 |

**Supplementary Figure S1. Contextual-model association of APS with FLI; shading shows 95% CI.**

**Supplementary Figure S2. Contextual multinomial APS associations with FLI categories (reference: FLI <30).**

**Supplementary Figure S3. FLI APS slopes across Townsend deprivation levels.**

**Supplementary Figure S4. FLI secondary analyses.**
